# Transmigration of Melanoma Cells through the Blood-Brain Barrier: Role of Endothelial Tight Junctions and Melanoma-Released Serine Proteases

**DOI:** 10.1371/journal.pone.0020758

**Published:** 2011-06-02

**Authors:** Csilla Fazakas, Imola Wilhelm, Péter Nagyőszi, Attila E. Farkas, János Haskó, Judit Molnár, Hannelore Bauer, Hans-Christian Bauer, Ferhan Ayaydin, Ngo Thi Khue Dung, László Siklós, István A. Krizbai

**Affiliations:** 1 Institute of Biophysics, Biological Research Center, Szeged, Hungary; 2 Department of Organismic Biology, University of Salzburg, Salzburg, Austria; 3 Laboratory of Cellular Imaging, Biological Research Center, Szeged, Hungary; University of New South Wales, Australia

## Abstract

Malignant melanoma represents the third common cause of brain metastasis, having the highest propensity to metastasize to the brain of all primary neoplasms in adults. Since the central nervous system lacks a lymphatic system, the only possibility for melanoma cells to reach the brain is via the blood stream and the blood-brain barrier. Despite the great clinical importance, mechanisms of transmigration of melanoma cells through the blood-brain barrier are incompletely understood. In order to investigate this question we have used an *in vitro* experimental setup based on the culture of cerebral endothelial cells (CECs) and the A2058 and B16/F10 melanoma cell lines, respectively. Melanoma cells were able to adhere to confluent brain endothelial cells, a process followed by elimination of protrusions and transmigration from the luminal to the basolateral side of the endothelial monolayers. The transmigration process of certain cells was accelerated when they were able to use the routes preformed by previously transmigrated melanoma cells. After migrating through the endothelial monolayer several melanoma cells continued their movement beneath the endothelial cell layer. Melanoma cells coming in contact with brain endothelial cells disrupted the tight and adherens junctions of CECs and used (at least partially) the paracellular transmigration pathway. During this process melanoma cells produced and released large amounts of proteolytic enzymes, mainly gelatinolytic serine proteases, including seprase. The serine protease inhibitor Pefabloc® was able to decrease to 44–55% the number of melanoma cells migrating through CECs. Our results suggest that release of serine proteases by melanoma cells and disintegration of the interendothelial junctional complex are main steps in the formation of brain metastases in malignant melanoma.

## Introduction

Malignant melanoma is the third common cause of brain metastasis behind lung and breast cancer, having the highest propensity to metastasize to the brain of all primary neoplasms in adults. Autopsy data indicate a prevalence of 55–75% of brain metastasis in melanoma. Brain metastases contribute to death in nearly 95% of patients with a median survival of less than one year (for review see: [1], [2]).

Since the central nervous system (CNS) lacks a lymphatic system, tumor cells can only reach the brain parenchyma by hematogenous metastasis formation. During this process metastatic cells need to traverse brain endothelial cells which in turn form the morphological basis of the blood-brain barrier (BBB). The BBB is a complex system with the main function of regulating the entry of blood-borne substances into the brain and thus maintaining the homeostasis of the CNS. Cerebral endothelial cells (CECs) – coming in contact with pericytes and astrocytes – form a single cell layer lining the blood vessels, and are sealed with a continuous belt of tight junctions (TJs) (for review see: [3]). TJs regulate the paracellular permeability of the endothelial layer and are composed of transmembrane proteins, including occludin, claudins and junctional adhesion molecules, and cytoplasmic plaque proteins which comprise zonula occludens proteins (ZO-1, ZO-2) and associated molecules (for review see: [4]). Development and maintenance of tight junctions is supported by adherens junctions (AJs) which are located basolaterally to TJs and also form a continuous line along cell-cell boundaries. AJs are also composed of transmembrane proteins (cadherins) and cytoplasmic proteins (catenins).

The process of transendothelial migration of tumor cells is largely uncharacterized, and much of our knowledge comes from endothelial cells of non-cerebral origin, which do not present the special BBB phenotype. Different cell surface and adhesion molecules, proteolytic enzymes and signaling pathways have been shown to facilitate invasive and migratory capacities of melanoma cells and their transfer through endothelial barriers. These include cadherins [5], P-glycoprotein [6] and the Rho-ROCK/Rac system [7]–[9].

However, very few experimental data are available about the interaction of melanoma cells with brain endothelial cells. It has been shown that the ability of melanoma cells to cross the BBB in vitro was correlated with their melanotransferrin expression levels at the cell surface [10]. Moreover, the fibrinolytic system was shown to play an important role [11]. Key steps in formation of melanoma brain metastasis were determined by in vivo real-time imaging and proved to be the following: arrest at vascular branch points, extravasation, persistent close contacts to microvessels and perivascular growth by vessel cooption [12].

In physiological conditions the endothelial barrier in the brain is so tight that not even ions can freely pass from one side to the other, therefore it is of special interest to elucidate how metastatic cells can migrate through the BBB. Moreover, taking into account the very poor prognosis and the limited therapeutical possibilities, it is of primordial importance to prevent melanoma brain metastasis, and therefore to understand the interaction between melanoma cells and CECs.

## Results

### Transmigration of melanoma cells through the *in vitro* blood-brain barrier model

In order to study the routes and mechanisms of transendothelial migration of melanoma cells we have developed an in vitro model system based on the culture of cerebral endothelial cells (primary brain endothelial cells isolated from rat: RBECs and a human cerebral microvascular endothelial cell line: hCMEC/D3, shortly D3) and two melanoma cell lines (the human A2058 and the murine B16/F10).

**Figure 1:**
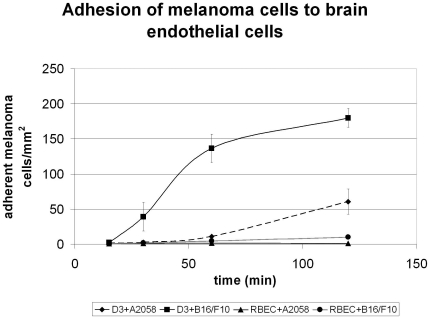
Adhesion of melanoma cells to brain endothelial cells. Fluorescently labeled melanoma cells (A2058 or B16/F10) (2.5·10^4^/cm^2^) were plated onto confluent CECs (RBEC or D3) and left for different time intervals. After washing of non-adherent cells, attached melanoma cells were counted.

We have observed that A2058 and B16/F10 melanoma cells were able to adhere to RBEC or D3 monolayers in a time dependent manner (Fig. 1). Melanoma cells started to attach to cerebral endothelial cells already after 15 min, process accelerated between 30–120 min. When parallely the same number of A2058 or B16/F10 cells was plated onto confluent brain endothelial monolayers, B16/F10 cells attached in a higher number to the endothelium than A2058 cells. Comparing the two endothelial monolayers, both melanoma cell types adhered much easier to the human cell line than to primary rat cells.

We have constructed a transmigration experimental setup (described in details in the Material and methods section) which consists of brain endothelial cells cultured on large pore size (8 µm) filter inserts. In our experience smaller pore sizes (including 3 µm) restrict the majority of tumor cells migrated through the endothelial cell layer to migrate onto the lower surface of the filter (not shown). In all cases we have verified the confluency of control endothelial monolayers by TEER measurements both at the beginning and at the end of the experiments and by immunofluorescence stainings of tight junction proteins. Fluorescently labeled melanoma cells were plated onto the apical side of the endothelial monolayer in a ratio of 9·10^4^ melanoma cells/cm^2^, which is lower than the number of tumor cells used in the literature in similar assays [13], [14]. We have also verified that melanoma cells labeled with Oregon Green® 488 carboxylic acid diacetate succinimidyl ester (shortly: OG) or CellTracker™ Blue did not stain the endothelial cells, not even after fixation. In our experience this was not always true when Hoechst 33342 was used. In the 5 h time frame proliferation of both cell lines was very low (not shown) and therefore did not affect the results of the transmigration assay. This setup makes possible the quantitative analysis of the transendothelial migration of tumor cells.

**Figure 2:**
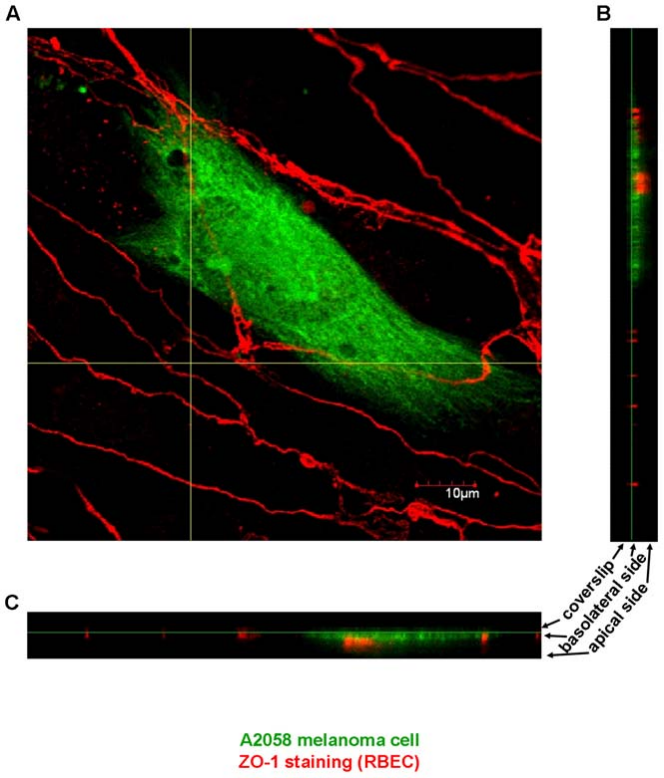
Migration of melanoma cells from the apical to the basolateral side of CECs. RBECs were grown on glass coverslips. A2058 melanoma cells labeled with OG were plated onto confluent endothelial monolayers. After 5 h cells were washed, fixed and tight junctions were stained with anti-ZO-1 antibody (red). Samples were analysed by confocal laser scanning microscopy. A: xy-stack at the level of the green lines on B and C. B, C: z-stacks along the vertical and horizontal line, respectively on A.

Both melanoma cell lines were able to cross the endothelial cell layer and migrate through the pores of the filter. Melanoma cells tended to accumulate on the lower side of the filter, where they could be easily counted. Transmigration of melanoma cells through brain endothelial cells could be visualized by z-stack laser confocal microscopy images as well. Endothelial cells were grown on coverslips and melanoma cells were plated onto the apical (luminal) side of the monolayer (“above” endothelial cells). Melanoma cells reaching the basolateral side of endothelial cells could be observed closer to the coverslip than the tight junctions of endothelial cells (“beneath” endothelial cells) (Fig. 2).

Performing time-lapse video (Video S1 and S2) we have observed that melanoma cells plated on a confluent brain endothelial monolayer started to eliminate protrusions at about 15–30 min after coming in contact with the endothelial cells. After about 60–120 min some melanoma cells started to change their morphology, becoming flattened and elongated, and completed the transmigration process in 15–30 min (S1 cell no.: 1 and 2a, S2 cell no.: 2 and 3). Other melanoma cells were not able to migrate through the endothelial monolayer in a 3–6 h time frame. Certain tumor cells covered several hundred micrometer long distances, stopping in different places on the apical side of the endothelial cell layer, without performing a successful transmigration (S2 cell no.: 1). We observed that the transmigration process of certain cells was accelerated when they were able to use the routes preformed by previously transmigrated melanoma cells (S1 cell no.: 3). Moreover, after migrating through the endothelial monolayer, melanoma cells did not remain in close proximity to the pore of transmigration but continued their movement beneath the endothelial cell layer (S1 cell no.: 1, S2 cell no.: 2 and 3).

**Figure 3:**
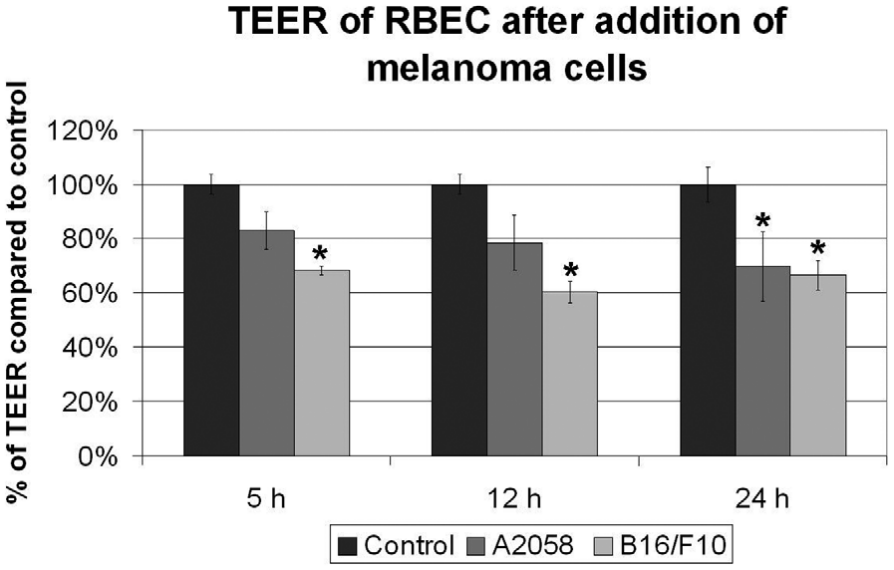
Changes in the transendothelial electrical resistance in the presence of melanoma cells. RBECs were grown on semipermeable filters with 0.4 µm pore size. TEER was followed using the CellZscope system. N = 2, * = P<0.05 as assessed by ANOVA and Bonferroni's post hoc test.

**Figure 4:**
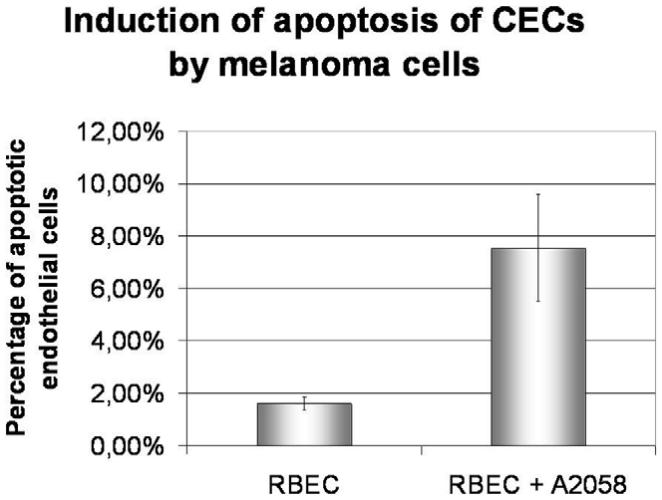
Induction of endothelial apoptosis by melanoma cells. RBECs were cultured on glass coverslips. A2058 melanoma cells labeled with CellTracker™ Blue were plated onto confluent endothelial monolayers. After 24 h cells were washed and fixed. Tight junctions were stained with anti-ZO-1 antibody (marker of endothelial cells), while apoptotic cells were visualized using anti-cleaved caspase-3 antibody. Apoptotic endothelial cells were counted. N = 2, * = P<0.05 as assessed by Student's t-test.

In order to investigate the possible effects of melanoma cells on the integrity of the barrier, we have measured the transendothelial electrical resistance (TEER) of CECs (Fig. 3). A2058 cells reduced the resistance of the endothelium in a time dependent manner, the decrease reaching 30% at 24 h; while B16/F10 melanoma cells induced a statistically significant decrease in TEER already at 5 h. Moreover, melanoma cells induced a 4.7 times increase in the number of apoptotic brain endothelial cells (from 1.7% in the absence of A2058 cells to 7.54%) as visualized by anti-cleaved caspase-3 staining (Fig. 4). No difference in the number of apoptotic melanoma cells was observed in the presence or absence of CECs (not shown).

### Paracellular transmigration of melanoma cells through the BBB

**Figure 5:**
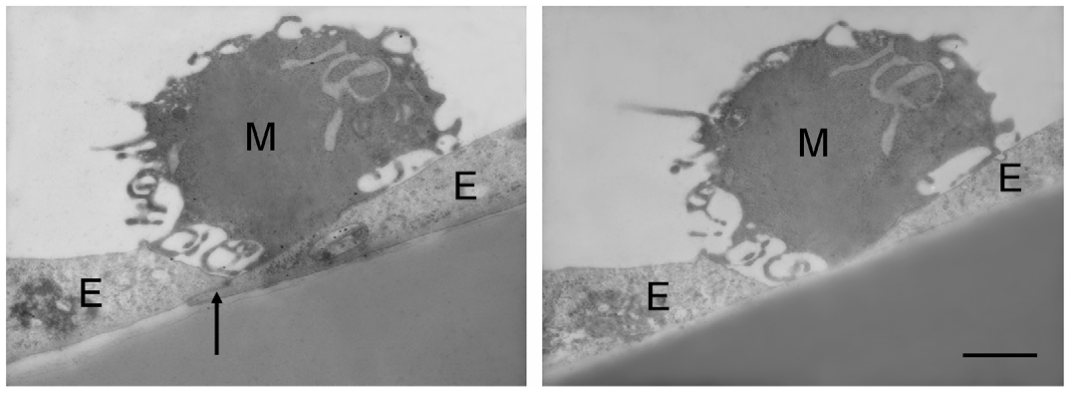
Adhesion of melanoma cells to interendothelial junctions. RBECs were grown on 8 µm pore size filter inserts. After reaching confluency A2058 cells were plated into the upper chamber and left for 5 h. Samples were fixed and 60–70 nm sections were prepared for electron microscopy. Arrow indicates interendothelial cell contact site. M = melanoma cell, E = endothelial cell. Scale bar = 10 µm.

It is well documented that leukocytes are able to use both the paracellular (through the interendothelial junctions) and the transcellular (through individual endothelial cells) transmigration pathways (for review see: [15], [16]). However, much less is known about the routes of transendothelial migration of tumor cells. Since the drop in the transendothelial electrical resistance of the cells indicated a damage of the tight junctions, in our experiments we have focused on the investigation of the paracellular migration of melanoma cells. Moreover, on sections examined by transmission electron microscopy we have found melanoma cells attached to the junctional sites of endothelial cells, raising the possibility that melanoma cells tended to migrate between two endothelial cells (Fig. 5).

**Figure 6:**
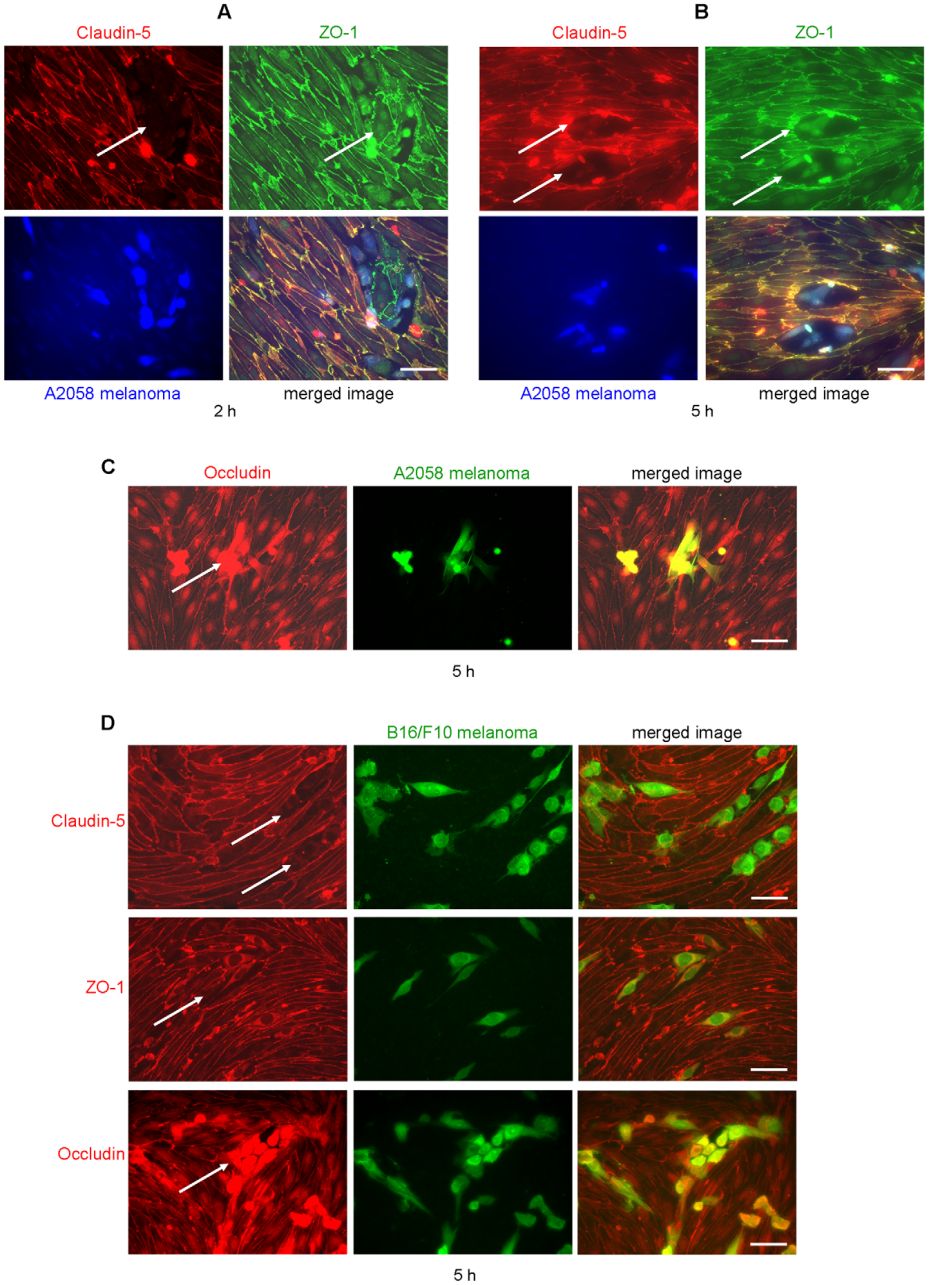
Disruption of interendothelial tight junctions induced by melanoma cells. Fluorescently labeled (A, B: CellTracker™ Blue, C, D: OG) melanoma cells (A, B, C: A2058 or D: B16/F10) were plated onto confluent brain endothelial cells (RBEC) and left for 2 (A) or 5 h (B–D). After washing of non-attached melanoma cells samples were fixed and stained for claudin-5, ZO-1 or occludin. Arrows indicate sites of disrupted junctions. Scale bar = 50 µm.

Therefore we have examined changes in the localization and expression of the main tight junction proteins. Fig. 6 shows that melanoma cells tended to adhere to brain endothelial cells in small clusters and were able to disrupt the continuous membrane staining of transmembrane TJ proteins (occludin and claudin-5), and the cytoplasmic plaque protein ZO-1. The same changes were observed in case of the adherens junction protein β-catenin (not shown). At 2 h co-culture of melanoma cells with brain endothelial monolayers claudin-5 disappeared totally from the region where melanoma clusters adhered to endothelial cells (Fig. 6 A first panel). At the same place ZO-1 staining was only partly disrupted (Fig. 6 A second panel). This suggests that the first affected TJ proteins were transmembrane proteins. At 5 h after plating of melanoma cells onto CECs no difference was observed between the localization changes of transmembrane and plaque proteins of tight junctions. Small clusters of A2058 cells adhered to CECs induced the disappearance of claudin-5, ZO-1 (Fig. 6 B) and occludin (Fig. 6 C) from the intercellular contacts. We could observe the same phenomenon in case of B16/F10 cells (Fig. 6 D).

**Figure 7:**
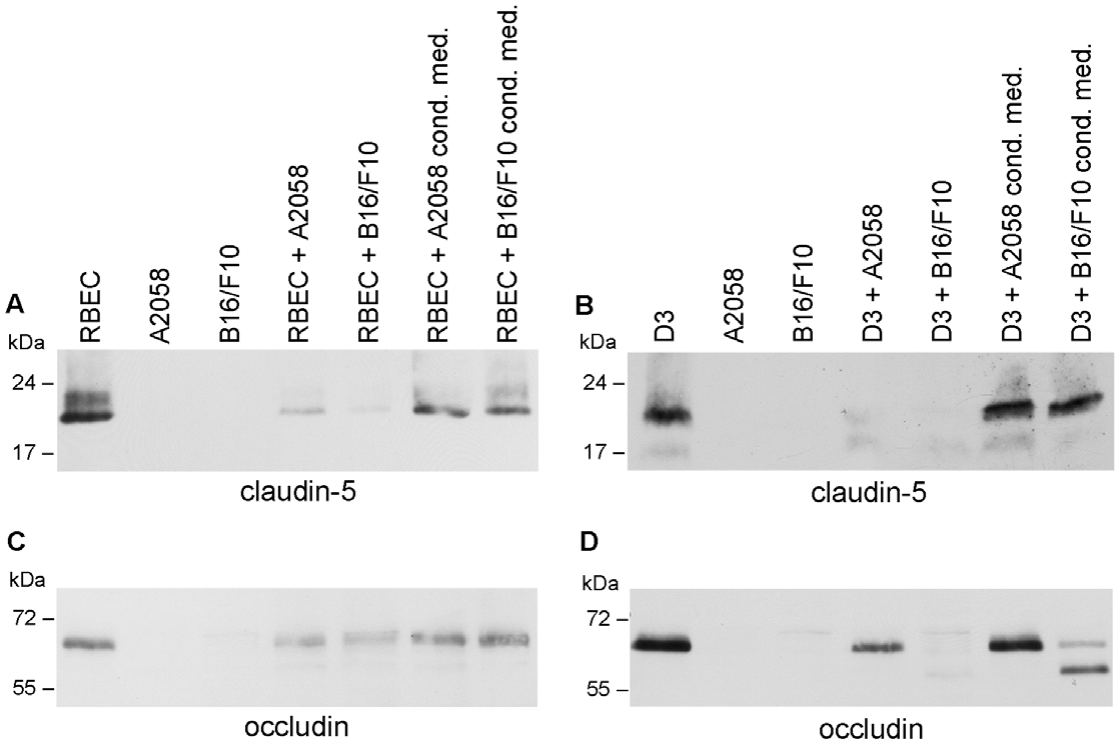
Changes in the total amount of endothelial junctional proteins in the presence of melanoma cells or melanoma-released factors. Melanoma cells or melanoma conditioned media were placed onto confluent brain endothelial cells (A, C: RBEC; B, D: D3) and the amount of claudin-5 (A, B) or occludin (C, D) was assessed by Western-blot analysis.

These results suggest that melanoma cells are able to take the paracellular pathway of transmigration. This was also supported by our Western-blot experiments (Fig. 7). As expected, neither A2058 nor B16/F10 cells expressed claudin-5 or occludin, which were present in both RBEC and D3 brain endothelial cells. The claudin-5 signal was strongly decreased by both melanoma cell lines in RBECs (Fig. 7 A) and disappeared completely from D3 cells (Fig. 7 B), while melanoma conditioned media induced a less pronounced effect. Similar tendencies could be observed in case of occludin (Fig. 7 C, D), where the degradation products could also be visualized in D3 cells in the presence of B16/F10 cells or conditioned medium (Fig. 7 D).

Our data unequivocally show that melanoma cells are able to disrupt the junctions of CECs and transmigrate through the paracellular pathway. However, these results cannot exclude the possibility that melanoma cells take the transcellular pathway as well.

### Role of serine proteases in the transendothelial migration of melanoma cells

**Figure 8:**
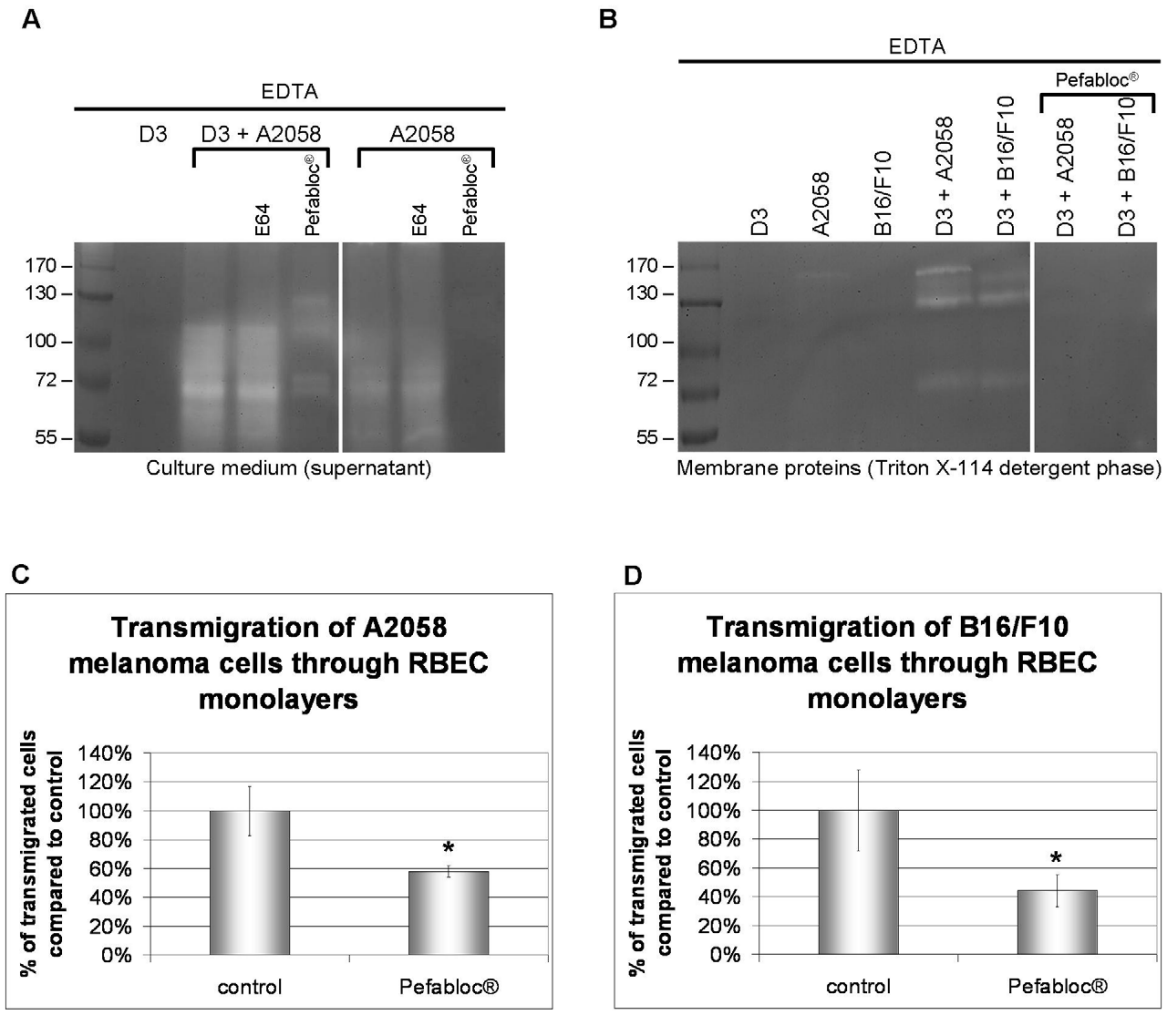
Role of gelatinolytic serine proteases produced by melanoma cells. A, B: Melanoma cells were plated onto confluent monolayers of cerebral endothelial cells or into empty culture dishes in serum-free medium in the presence or absence of E64 or Pefabloc® and left for 5 h. Culture media were collected and cells were lysed in Triton X-114 containing buffer. Samples were electrophoresed in non-denaturing conditions and the gels were incubated in EDTA-containing buffer for 2 days. Proteolytic bands of culture media (A) or cell lysates (B) were visualized by Coomassie blue staining. C, D: RBEC were grown until confluency on 8 µm pore size filter inserts. Fluorescently labeled melanoma cells (C: A2058, D: B16/F10) were plated into the upper chamber in the presence or absence of Pefabloc® and left for 5 h. Cells from the upper chamber were removed using a cotton swab, and melanoma cells migrated through the endothelial cell layer and the pores of the filter were counted. N = 3, * = P<0.05 as assessed by Student's t-test.

As a next step we have investigated the production of proteases by A2058 and B16/F10 melanoma cells in the presence and absence of brain endothelial cells by gelatin zymography. Proteolytic enzymes both released in the culture medium (Fig. 8 A) and their membrane bound forms (Triton X-114 fraction) (Fig. 8 B) were analyzed. We have observed that both melanoma cell lines expressed several gelatinolytic proteases and the amount of these was increased when melanoma cells were plated onto brain endothelial cells. Unexpectedly, the gelatinolytic bands did not disappear in the presence of the matrix metalloproteinase inhibitor EDTA (Fig. 8 A, B). Addition of E-64, an irreversible, potent and highly selective cysteine protease inhibitor had no effect on the release of gelatinolytic enzymes. However, addition of Pefabloc®, an irreversible serine protease inhibitor, induced the almost complete disappearance of the proteolytic bands from both the supernatant and the cell lysate fraction. This indicates that gelatinolytic serine proteases are produced by melanoma cells in large amounts especially when coming in contact with endothelial cells.

We have also observed that presence of Pefabloc® significantly reduced the transendothelial migration rate of both A2058 and B16/F10 melanoma cells (to 58±4% in case of A2058 and to 44±11% in case of B16/F10) (Fig. 8 C, D). Migration of melanoma cells in the absence of endothelial cells was not affected by Pefabloc® as assessed by the wound assay (not shown).

**Figure 9:**
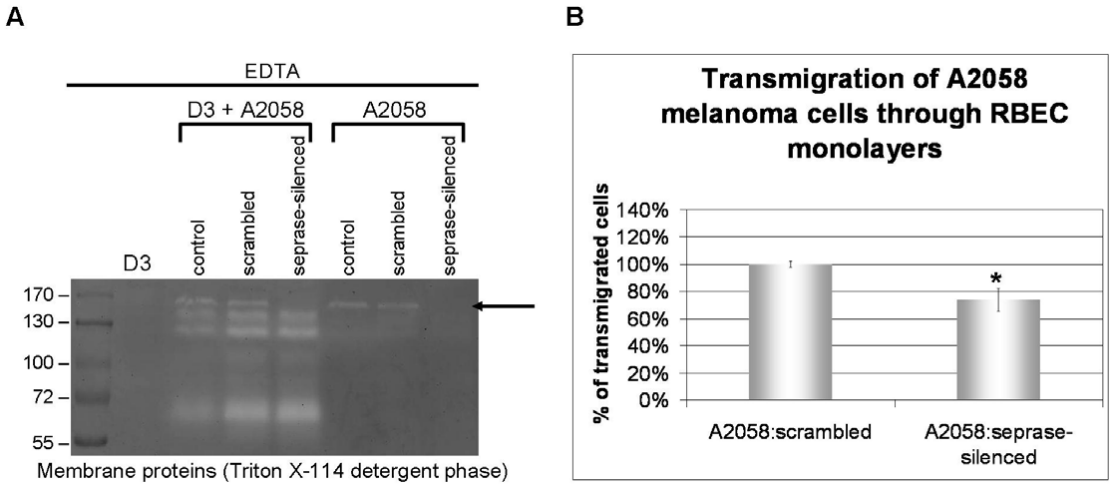
Role of seprase in the transmigration of A2058 cells through brain endothelial cells. A: Seprase was silenced in A2058 cells (fold change = 0.09 compared to scrambled RNA-transfected cells, determined by real-time PCR). Melanoma cells were plated onto confluent monolayers of cerebral endothelial cells or into empty culture dishes in serum-free medium and left for 5 h. Cells were lysed in Triton X-114 containing buffer. Zymography was performed in the presence of EDTA. Arrow indicates seprase. B: Seprase-silenced or scrambled RNA-transfected cells were plated onto confluent RBECs grown on 8 µm pore size filter inserts. Transmigration assay was performed and melanoma cells reaching the bottom of the filter inserts were counted. N = 3, * = P<0.05 as assessed by Student's t-test.

As a next step we wanted to identify which serine proteases are expressed by melanoma cells. One of the most well characterized serine protease with gelatinolytic activity is seprase, which has already been shown to be activated in tumors [17]. As indicated on Fig. 8 B, A2058 but not B16/F10 melanoma cells express a 170 kDa gelatinolytic membrane-bound serine protease. After silencing of the seprase gene the 170 kDa gelatinolytic band disappeared from the zymography gel (Fig. 9 A). Therefore, we could identify seprase as one of the gelatinolytic serine proteases expressed by A2058 cells. Silencing of seprase in A2058 melanoma cells induced a more than 20% decrease in the number of cells transmigrated through brain endothelial cells (Fig. 9 B). These data suggest that seprase plays an important, but not a unique role among proteases involved in the transmigration of melanoma cells through the BBB.

## Discussion

The CNS is a common site for melanoma metastasis. Development of brain metastases is of very poor prognosis despite extensive therapeutical efforts. Therefore, it is very important to understand the mechanisms of migration of tumor cells through the BBB, which could eventually help to develop preventive strategies against the formation of melanoma brain metastasis.

In order to study the mechanisms of transendothelial migration of melanoma cells, we have developed an in vitro model system. Due to the lower needs of experimental animals and lower costs, in vitro models are becoming more and more used prior to or parallel with in vivo models. Moreover, since rodents rarely develop cerebral metastases of melanoma, inoculation of tumor cells into the systemic circulation or into the brain is used to study this question. These in vivo systems however, are not suitable to study specific mechanisms regarding the migration of tumor cells through the BBB. The in vitro approach makes possible the detailed analysis of the mechanisms of transmigration of melanoma cells through brain endothelial monolayers, which cannot be readily approached in vivo because of the complexity of the system. Similar systems have been widely used to study the transmigration of leukocytes through endothelial barriers [18], [19].

The in vitro BBB model used in our experiments is based on the culture of primary rat brain endothelial cells (RBECs) or the human cerebral endothelial cell line hCMEC/D3 (D3). RBECs in our culture system maintain the main characteristics of the brain endothelium in vivo, such as expression of von Willebrand factor, presence of a continuous line of tight junctions, high transendothelial electrical resistance (TEER) and low permeability values and high activity of P-glycoprotein. However, the fact that rodents develop in very rare instances spontaneous melanoma brain metastases [20] points towards significant differences between the human and animal disease. This makes necessary the use of human endothelial cells despite their inferior permeability characteristics. Moreover, it has been shown that human melanoma cells injected into mice give spontaneous CNS metastasis [12], [21], therefore a xenogeneic model can also be accepted. The hCMEC/D3 cell line [22] is the most well characterized human brain endothelial cell line, which has been widely used as a human BBB model [23], [24].

We have used the A2058 cell line, which has been shown to produce brain metastasis when injected into immunodeficient mice [12]. We have also used murine B16/F10 cells, which initially were adapted to form lung metastasis; however, they are able to form brain metastases as well [25], [26].

Our results show that melanoma cells attached in a higher number to D3 cells than to RBECs. This might be due to the difference in the human and rat adhesion molecules, but also to the lower tightness of the barrier formed by the human cell line.

Our time-lapse video suggests that transmigrated melanoma cells tended to attract other melanoma cells to migrate through the endothelial cell layer at the same site. We could also visualize that transmigrated melanoma cells moved along the basolateral side of the endothelial monolayer. This is in accordance with the observations of Lu et al. [27] who described that breast cancer cells injected intracardially into mice extravasated into the brain and aligned themselves along the blood vessels, on the basolateral side of endothelial cells, suggesting that the tumor cells migrated along the vasculature. This “pericyte-like” position of transmigrated tumor cells was also observed by Kienast et al. [12] using in vivo multiphoton laser scanning microscopy. They have described that melanoma and lung cancer cells could only proliferate in the brain if they maintained a direct contact to the abluminal side of endothelial cells of cerebral capillaries. These results might raise the possibility of a hiding mechanism of tumor cells behind the defense lines (especially the MDR-MRP systems) of the BBB.

During transmigration melanoma cells damaged the integrity of the endothelial monolayer, which is supported by the decrease of TEER and the presence of apoptotic endothelial cells. These observations are in accordance with previous results showing that – in contrast to leukocytes – tumor cells do not leave the endothelium intact after diapedesis [28].

Regarding the routes of transmigration, there are two theoretical possibilities, which have been intensively studied in the case of leukocytes: the paracellular pathway (through the interendothelial junctions) and the transcellular one (through single endothelial cells). A large number of in vitro and in vivo studies have demonstrated that leukocyte diapedesis can occur either by forming a paracellular gap or via the formation of a transcellular pore (for review see: [15], [16]). The phases and molecular mechanisms involved in the extravasation of leukocytes have also been largely characterized. Since the CNS is an immunologically privileged site, transendothelial migration of leukocytes through the BBB is practically limited to neuroinflammation. Mononuclear cells can traverse the inflamed BBB both through the tight junctions (paracellular pathway) [29] and transcellularly [30].

However, much less is known about the extravasation of tumor cells (for review see: [28]). It has been shown that – similarly to leukocytes – breast cancer cells are able to use both the para- and the transcellular transmigration routes [31]. Here we show that melanoma cells are able to disrupt interendothelial junctions and to migrate through the paracellular pathway.

One of the key steps of successful metastasis formation is the proteolytic degradation of the extracellular matrix. This process is important not only during the invasion of the surrounding tissue by the primary tumor and the step of intravasation, but also during extravasation. Moreover, tight junction proteins are also targets of proteolytic degradation [29]. The plasminogen/plasmin system has been shown to facilitate transmigration of melanoma cells through brain endothelial cells both in vivo and in vitro [11]. Melanoma cells are able to express several types of proteases including uPA [32], seprase [33], [34] and matrix metalloproteinases [35]–[37] which have been shown to promote their invasiveness.

Our results show that during the transendothelial migration process melanoma cells express on their membranes and release large amounts of proteases with gelatinolytic activity. Much to our surprise the majority of these enzymes proved to be not matrix metalloproteinases, since addition of EDTA to the incubation buffer did not decrease the number and intensity of the proteolytic bands. In contrast, after addition of the irreversible serine protease inhibitor Pefabloc® to the culture medium the gelatinolytic bands almost completely disappeared and the number of melanoma cells migrated through the brain endothelial monolayer decreased significantly.

We have examined whether among these gelatinolytic serine proteases seprase and/or its degradation products are present. Seprase (surface expressed protease) or FAPα (fibroblast activation protein α) is a type II transmembrane glycoprotein, originally identified in LOX melanoma cells [34], contributing to the invasiveness of melanoma and carcinoma cells (for review see: [38]). It has two types of EDTA-resistant protease activities: dipeptidyl peptidase and a 170 kDa gelatinase activity. Seprase has been identified as a potential marker protease of invasiveness, localized on invadopodia of malignant melanoma cells [33]. Seprase shares homology with dipeptidyl peptidase IV (DPPIV), however, this latter is expressed by normal melanocytes, epithelial and other cells, while seprase is characteristic for tumor and proliferating mesenchymal cells [39], [40]. Using PCR we have found that human brain endothelial D3 cells express DPPIV, but do not express seprase, while A2058 human melanoma cells express seprase, but not DPPIV (not shown). By gelatin zymography coupled with gene silencing we have shown that A2058, but not B16/F10 cells express seprase in the Triton X-114-extractable membrane fraction. However, several other gelatinolytic serine proteases were expressed by both melanoma cell lines which did not disappear after seprase-silencing. Accordingly, silencing of seprase reduced to a lesser extent the number of transendothelially migrated melanoma cells compared to Pefabloc®. The identity of these enzymes still needs to be elucidated.

In conclusion, our in vitro results suggest that during brain metastasis formation melanoma cells damage the integrity of the BBB by inducing apoptosis of endothelial cells and by disrupting the continuity of the tight junctions. Transmigrating melanoma cells are able to use the paracellular pathway to overcome the cerebral endothelial monolayer and seem to be able to hide behind the defense lines of the BBB. During their transendothelial migration melanoma cells produce serine proteases capable of degrading the components of the basement membrane of capillaries, and inhibition of these proteases can significantly reduce the number of extravasating melanoma cells.

## Materials and Methods

### Cell culture

A2058 human melanoma cells (obtained from the European Collection of Cell Cultures) were maintained in MEM (Sigma) supplemented with 5% FBS (Lonza) and Glutamax (Invitrogen). B16/F10 murine melanoma cells were kept in RPMI medium (Sigma) supplemented with 5% FBS (Lonza) and Glutamax. The hCMEC/D3 [22] human cerebral endothelial cells (shortly D3) were grown on rat tail collagen-coated dishes in EBM-2 medium (Lonza) supplemented with EGM-2 Bullet Kit (Lonza) and 2.5% FBS (Sigma).

Primary rat brain endothelial cells (RBECs) were isolated from 2-week old rats, as described previously [41], [42]. Briefly, after removal of meninges cerebral cortices were cut into small pieces and digested with 1 mg/ml collagenase type 2 (Sigma) for 75 min at 37°C. After separation of myelin by centrifugation in 20% BSA, a second digestion was performed with 1 mg/ml collagenase/dispase (Roche) for 50 min at 37°C. Microvessel fragments were collected after 10 min 1000•g centrifugation on Percoll (Sigma) gradient, and plated onto fibronectin/collagen-coated dishes. Endothelial cells growing out of the microvessels were cultured in DMEM/F12 (Invitrogen), 10% plasma-derived serum (PDS, First Link) and growth factors. In the first two days, 4 µg/ml puromycin was added to remove contaminating cells.

Isolation of primary cerebral endothelial cells was carried out in strict accordance with the national and international recommendations for the care and use of laboratory animals. The protocol was approved by the Regional Animal Health and Food Control Station of Csongrád County (Permit Number: XVI./03839/001/2006). All efforts were made to minimize suffering.

### Time-lapse video imaging

D3 cells were grown in 6 cm culture dishes. After reaching confluency 10^6^ A2058 cells were plated onto the endothelial cell layer and phase contrast images were taken every 5 min using a digital camera (Spot RT KE, Diagnostic Instruments) connected to the microscope (Nikon Eclipse TE2000U). Experiments were performed at 37°C. Serial images were converted to video stream with VirtualDub version 1.9.10. Xvid MPEG4 video codec version 1.2.2 was used for video compression.

### Adhesion experiments

Brain endothelial cells (RBEC or D3) were grown until confluency in 24-well plates. Melanoma cells (A2058 or B16/F10) were fluorescently labeled using Oregon Green® 488 carboxylic acid diacetate succinimidyl ester (shortly: OG, Invitrogen) using the protocol supplied by the manufacturer. 5·10^4^ melanoma cells/well were loaded onto the endothelial cells in serum-free medium and left for different time intervals. Non-attached cells were washed and remaining cells were fixed using ethanol/acetic acid (95/5) at −20°C for 5 min. Melanoma cells adhered to endothelial cells were photographed and counted using the Image-Pro Plus software.

### Transmigration experimental setup

For transmigration experiments primary RBECs were gently trypsinized and passed onto fibronectin/collagen-coated filter inserts (8 µm pore size, 1.13 cm^2^, Millipore) which were placed in 12-well plates. After reaching confluency, endothelial cells were supplemented with 550 nM hydrocortisone, 250 µM CPT-cAMP (Sigma) and 17.5 µM RO-201724 (Roche) from the apical side and astrocyte conditioned medium from the basolateral side for 24 h in order to tighten the junctions. This way we could achieve high transendothelial electrical resistance values.

10^5^ OG-labeled melanoma cells were plated into the upper compartment onto the endothelial monolayer in serum-free medium. The lower compartment was loaded with serum-free medium containing 100 µg/ml type I collagen. Pefabloc® was added in a concentration of 200 µM to both the apical and basolateral side. Cells were left for 5 h, followed by fixation with ethanol/acetic acid. Cells from the upper compartment were removed with a cotton swab and melanoma cells migrated through the endothelial monolayer and the pores of the filter were counted.

### Immunofluorescence and laser confocal microscopy

RBECs (P0 or P1) were cultured until confluency on collagen/fibronectin-coated glass coverslips. Melanoma cells (A2058 or B16/F10) were fluorescently labeled using CellTracker™ Blue CMAC (Invitrogen) or OG and plated onto the endothelial monolayer. After 5 h cells were washed and fixed with ethanol/acetic acid. After blocking with 3% BSA (Sigma) for 30 min, coverslips were incubated with primary antibodies against occludin, claudin-5 or ZO-1 (Zymed) or cleaved caspase-3 (Cell Signaling). The staining was visualized using Cy3- or Cy5-conjugated secondary antibodies. Coverslips were mounted in anti-fading embedding medium (Biomeda) and the distribution of the signal was studied using a fluorescence microscope.

Three dimensional optical sectioning was performed using an Olympus Fluoview FV1000 confocal laser scanning microscope. Microscope configuration was the following: Objective lens: UPLSAPO 60 x oil immersion objective (N.A:1.35); XY scanning dimensions: 512·512 pixels with 0.094 µm/pixel; Z dimension: 15 sections with 0.47 µm/slice; sampling speed: 2 µs/pixel; confocal aperture: 152 µm; zoom: 4.4 x; scanning mode: sequential unidirectional; excitation: 488 nm (OG) and 543 nm (Cy3); laser transmissivity: 9% and 20% were used for OG and Cy3, respectively; main dichroic beamsplitter: DM405/488/543; intermediate dichroic beamsplitter: SDM 560; OG was detected between 500–530 nm, Cy3 was detected between 555–655 nm with spectral detectors. Using the Olympus Fluoview software (version 1.7.2.2), OG and Cy3 images were pseudocolored green and red, respectively.

### Measurement of transendothelial electrical resistance (TEER)

RBECs were grown on collagen/fibronectin-coated semipermeable filters (0.4 µm pore size, 1.12 cm^2^, Costar Corning Transwell Clear). After reaching confluency, the endothelial monolayer was supplied with 550 nM hydrocortisone, 250 µM CPT-cAMP (Sigma) and 17.5 µM RO-201724 (Roche) and placed into the wells of the CellZscope® instrument (nanoAnalytics) containing astrocyte conditioned medium. After TEER had reached plateau, 10^5^ melanoma cells were plated into the apical chamber and TEER was followed for 24 h.

### Transmission electron microscopy

RBECs were cultured on 8 µm pore size filter inserts. 2·10^5^ A2058 melanoma cells were plated onto the endothelial monolayer and left for 5 h. After washing in PBS cells were fixed in 1% formaldehyde and 1% glutaraldehyde in 0.1 M phosphate buffer and postfixed in 1% osmium tetroxide in 0.1 M phosphate buffer. Fixed cells were dehydrated and embedded in Spurr resin. Thin sections were cut using a Leica ultramicrotome, contrasted with uranyl acetate and lead citrate and examined using a Zeiss 902 electron microscope.

### Western-blot analysis

Melanoma cells were plated onto confluent brain endothelial cells (RBEC or D3) in serum-free medium and left for 24 h. Cells were washed with PBS and scraped into ice-cold lysis buffer (20 mM Tris, 150 mM NaCl, 0.5% Triton X-100, 1% sodium deoxycholate, 0.1% sodium dodecyl sulphate, 1 mM sodium vanadate, 10 mM NaF, 1 mM EDTA, 1 mM Pefabloc®) and incubated on ice for 30 min. Lysates were clarified by centrifugation at 10,000•g for 10 min at 4°C. Proteins were electrophoresed and blotted onto PVDF (Millipore) or nitrocellulose (Whatman) membranes. Blocking the nonspecific binding capacity of the membranes was carried out at room temperature for 30 min in TBS-T containing either 5% casein (nonfat milk powder) or 3% BSA in the case of claudin-5. Anti-claudin-5 (Zymed) and anti-occludin (Transduction Laboratories) primary antibodies were used. After washing the membranes in TBS-T, blots were incubated with the secondary antibodies (Pierce) diluted in TBS-T. The immunoreaction was visualized using the Immobilon Western Chemiluminescent HRP Substrate (Millipore) on X-ray film (Agfa).

### Zymography

D3 cells were grown in 12-well plates. 2·10^5^ A2058 melanoma cells were plated onto the endothelial monolayer in serum-free medium and left for 5 h. Culture media were collected, clarified by centrifugation at 10,000·g for 10 min on 4°C and prepared in mercaptoethanol-free Laemmli buffer. Cells were washed and lysed in ice cold TBS containing 1.5% Triton X-114. Samples were centrifuged at 4°C and the supernatants were placed to 37°C for 5 min. After centrifugation at room temperature for 2 min the upper aqueous phase was thrown away and the membrane fraction was dissolved in mercaptoethanol-free Laemmli buffer. Samples were electrophoresed under non-denaturing conditions in a polyacrylamide gel containing 1.5 mg/ml gelatin. Gels were washed two times in 2.5% Triton X-100 and two times in water and incubated for two days at 37°C in a buffer containing 50 mM Tris pH = 7.4, 5 mM CaCl_2_, 1 mM ZnSO_4_, 1 mM MgCl_2_ and 0.2 M NaCl or 50 mM Tris pH = 7.4, 0.2 M NaCl and 5 mM EDTA. Gels were stained with Coomassie BBR-250 for 20 min and destained using 10% methanol and 10% acetic acid until the gelatinolytic bands became visible.

### Specific knockdown of seprase by RNA interference

Stealth™ siRNA duplex oligoribonucleotides were designed using Invitrogen BLOCK-iT™ RNAi designer and were purchased from Invitrogen. The sequences used were as follows: sense: 5′- AAGAAGUGUGUUACUUGCCAUCUAA-3′; antisense: 5′- UUAGAUGGCAAGUAACACACUUCUU -3′. As control non-targeting RNA we have used the following scrambled oligonucleotides: sense: 5′-GACGUAGAGAGAGUUCCGACAUACA-3′ and antisense: 5′-UGUAUGUCGGAACUCUCUCUACGUC-3′. Briefly, A2058 cells were plated at 50% confluency. Transfection of oligonucleotides was performed in OptiMEM medium containing 10 nM RNA and Lipofectamine™ RNAiMAX reagent (Invitrogen) following the manufacturer's instructions. After 8 h the medium was changed to regular culture medium. In order to increase the efficiency, a second transfection was performed the following day. Cells were used 24 h after the second transfection. At the same time transfection efficiency was analyzed by real-time PCR.

## Supporting Information

Video S1 **Time-lapse video imaging of transmigration of melanoma cells through brain endothelial monolayers.** A2058 cells were plated onto confluent D3 cultures. Phase contrast images were taken at every 5 min from the same regions and time-lapse videos were constructed. 1 s in the video file corresponds to 50 min in real time.
(AVI)

Video S2 **Time-lapse video imaging of transmigration of melanoma cells through brain endothelial monolayers.** A2058 cells were plated onto confluent D3 cultures. Phase contrast images were taken at every 5 min from the same regions and time-lapse videos were constructed. 1 s in the video file corresponds to 50 min in real time.
(AVI)
